# µCodes: A Universal Grid Platform for Microscale Mapping, Microscopy Navigation, and Multimodal Imaging

**DOI:** 10.1002/smll.202506183

**Published:** 2025-08-21

**Authors:** Aref Valipour, Jungmin Ha, Stephen N. Housley

**Affiliations:** ^1^ School of Biological Sciences Georgia Institute of Technology Atlanta GA 30332 USA; ^2^ School of Electrical and Computer Engineering Georgia Institute of Technology Atlanta GA 30332 USA; ^3^ Scheller College of Business Georgia Institute of Technology Atlanta GA 30332 USA; ^4^ George W. Woodruff School of Mechanical Engineering Georgia Institute of Technology Atlanta GA 30332 USA; ^5^ Institute for Bioengineering and Biosciences Georgia Institute of Technology Atlanta GA 30332 USA; ^6^ Winship Cancer Institute Emory University Atlanta GA 30322 USA

**Keywords:** µCodes, cell characterization, electron microscopy, imaging grids, microcodes, multi modal imaging

## Abstract

Different imaging modalities provide unique and valuable insights into biological sample characterizations. Integration of multimodal imaging techniques is essential for comprehensive biological sample analysis. µCodes, a microgrid system designed to enhance sample navigation, alignment, and co‐registration of images obtained from various optical and electron microscopy techniques, is presented. µCodes is a platform for tracking and locating targets or cells of interest across different imaging modalities with high precision. In this paper, the design, fabrication, and implementation of µCodes are demonstrated, emphasizing their utility in imaging workflows for biological samples.

## Graphical abstract

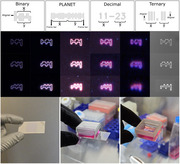

## 1 Introduction

Microscopy serves as an irreplaceable methodological backbone within biology and biomedical sciences. It provides a critical tool to investigate, characterize, and enumerate biological phenomena^[^ ^1^, ^2^, ^3^ ^]^ and is increasingly relied on to direct downstream assays such as genetic sequencing^[^ ^4^, ^5^, ^6^, ^7^ ^]^ and mass spectrometry.^[^ ^8^ ^]^ As the complexity of scientific questions increases, the demand for more sophisticated imaging approaches must evolve in tandem.^[^ ^3^, ^9^, ^10^ ^]^ Multimodal imaging, the process of applying multiple techniques toward the same sample, that each offer unique insights, e.g., optical, fluorescence, confocal, electron microscopy (EM), surface profiling is a widely used approach to meet this demand.^[^ ^11^, ^12^, ^13^, ^14^, ^15^, ^16^ ^]^ While multimodal imaging such as correlative microscopy, has the potential to increase inferential power, it depends on integrating data from disparate platforms, a process that faces significant challenges, many of which have yet to be overcome.^[^ ^1^ ^]^ A key bottleneck in multimodal workflows is the reliable navigation and re‐identification of specific regions or targets of interest, e.g., cells, across different instruments.^[^ ^1^ ^]^ Each microscopy system possesses unique characteristics, including imaging physics and conditions, optical configurations, coordinate systems, fields of view, and navigation mechanisms.^[^ ^17^ ^]^ These differences make following a target through sequential sample preparations and image acquisition steps laborious and prone to error.^[^ ^1^, ^17^, ^18^ ^]^ Adding to this difficulty, samples and common imaging substrates such as polymers, resins, or gels are susceptible to dimensional changes—swelling, shrinking, or warping—due to variations in temperature, humidity, atmospheric pressure, and/or exposure to solvents encountered during sample preparation or imaging protocols.^[^ ^19^, ^20^, ^21^ ^]^ Such substrate instability undermines the reliability of using sample features as fixed reference points.

Integrating imaging modalities into a single instrument is one solution but necessitates costly capital expenditures on new devices and often leads to compromises in performance relative to dedicated instrumentation.^[^ ^22^ ^]^ Alternatively, integrating data from disparate sources requires an exogenous reference frame applied through manual marking, e.g., via surface scratching^[^ ^23^ ^]^ or Focused Ion Beam,^[^ ^24^ ^]^ detection of random or pseudo‐random structures,^[^ ^25^ ^]^ e.g., gold nanoparticles deposited on the substrate,^[^ ^26^, ^27^ ^]^ post‐acquisition computational image registration,^[^ ^28^, ^29^, ^30^, ^31^, ^32^ ^]^ or a combination of these techniques.^[^ ^33^, ^34^ ^]^ In all cases, current techniques suffer from limitations in real‐time application, accuracy, reproducibility, or the ability to dynamically compensate for sample distortions.

Establishing a robust, instrument‐agnostic coordinate system is therefore crucial to streamline multimodal experiments, reduce localization time and effort, and potentially provide a normalization framework to counteract substrate distortions experienced during different experimental conditions. This capability is particularly vital for correlative studies requiring fast, high‐precision alignment, such as cross‐validating fluorescence microscopy data with high‐resolution EM.^[^ ^13^, ^25^ ^]^ Precise cell‐level correlation enables validation of cell behavior and antibody specificity, moving beyond tissue‐level assessments to investigate differential staining intensities, probe the mechanisms behind protein interactions, and potentially explain ambiguous results like false positives or co‐staining with multiple markers.^[^ ^35^ ^]^ Such precise tracking is also essential for studying rare events or cell populations, such as circulating tumor cells (CTCs), throughout complex experimental and analytical pipelines.^[^ ^36^ ^]^

To address these challenges, we present µCodes: a microscale engineered grid platform for robust navigation, mapping, and integration of multimodal imaging data at the single‐cell and population level. µCodes provide an unambiguous sample reference frame capable of identification and tracking precise sample locations. µCodes are adaptable and cross‐modality compatible with wide‐scale invariance (250‐fold scale invariance on our 80 µm‐footprint binary design with application ranging from 4X optical microscopy to 1000X SEM), enabling applications in optical‐, confocal‐, and electron‐microscopy. µCodes provide sufficient information to orient and direct sample navigation. Moreover, µCodes do not interfere with primary data collection. This system facilitates the precise tracking of specific biological targets, including rare cell populations such as circulating tumor cells (CTCs), through multiple characterization stages with minimal disruption. We demonstrate this spectrum of µCodes capabilities through a multimodal imaging and correlative microscopy study of cancer cells. We discuss the potential of µCodes to transform multimodal imaging workflows by providing a substrate‐side coordinate system that reduces the time and effort required to identify, locate, and follow targets of interest while also serving as a normalization vector for image processing techniques. The µCodes system represents an advancement in microscopy navigation that addresses the critical need for consistent reference points across the expanding landscape of multimodal imaging and correlative microscopy.

## 2 Results

### 2.1 Design Principles of µCodes

Correlative microscopy and multimodal imaging rely on the robust identification of targets of interest across disparate instrumentation and further depends on integrating distinct data sources, which could be compromised by a lack of an instrument agnostic, unambiguous sample reference frame. We reasoned that samples could be monitored by incorporating a scalable fiducial grid system, that precisely identifies substrate coordinates with uniquely defined symbols. By generating a sample holding substrate that contains an underlying grid of unique 3D geometric patterns and directly collecting or mounting cells or tissues of interest, integration of both the samples and patterns would be obligatory. During data collection, the patterns will be imaged together with the targets of interest and act as an encoded unique identifier to determine precise sample location. As each 3D geometric symbol is distinct but situated within a global symbol‐to‐symbol mapping scheme, reidentifying samples of interest across modalities, the direction and orientation of stage navigation, and data co‐registration could be automatically performed. To test this idea, we set out to develop a scalable spatial referencing system to serve as fiducial guides for seamless navigation across disparate imaging modalities and analytical techniques, would be biocompatible and not interfere with the collection of primary data.

**Figure 1:**
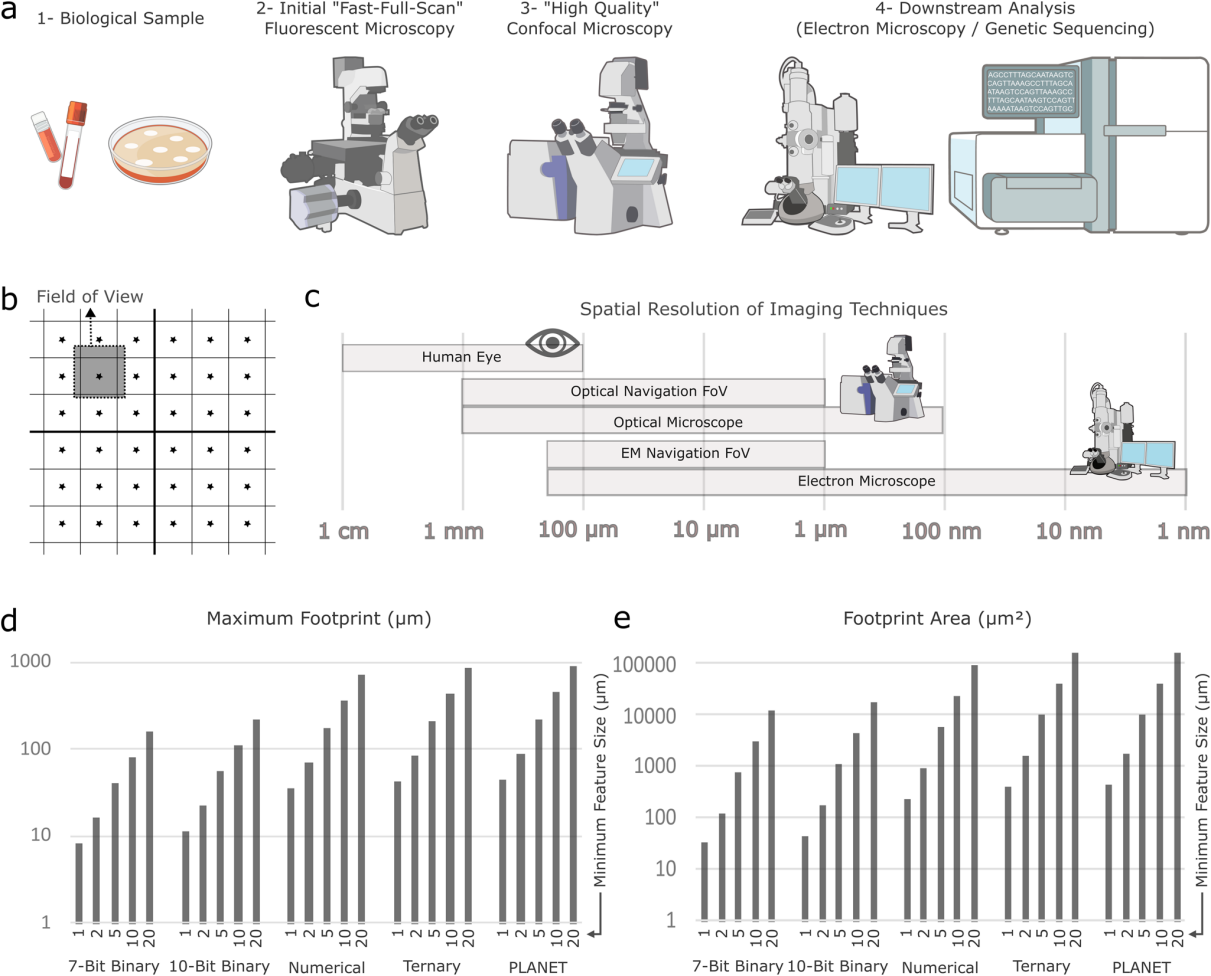
µCodes design and implementation. a) A sample process flow for analysis of a biological sample. As an example of the process, a biological sample such as a blood sample containing Circulating Tumor Cell would go through isolation/selection, immunofluorescent staining, high‐ and low‐quality optical imaging, and downstream analysis such as genetic sequencing or electron microscopy. As each of these steps either prepares the sample for the analysis or perform the analysis to gather the intended information, it is important to follow the target specimen throughout these processes. b) An illustration of µCodes design logic. µCodes are designed to have at least one µCodes per largest field of view of the bottleneck instrument. c) Examples of most popular imaging techniques and the size of their respective field of view. Navigation field of view (FoV) is defined as the largest field of view that the user navigates in, and is where the target sample, e.g., a cell, is comfortably distinguishable for the user. Optical microscopes include fluorescent, confocal microscopes, and instruments using optical microscopy, e.g., laser dissection and micromanipulator. Electron microscopes represent Scanning Electron Microscopes (SEMs), Transmission Electron Microscopes (TEMs), and their derivatives such as Focused Ion Beam. d) A comparison of the maximum dimension footprint of the µCodes with various designs and minimum features sizes. Binary µCodes are the most compact variation given any feature size. e) A comparison of the footprint area of the µCodes with various variations and minimum feature sizes. Instrument images available at https://bioart.niaid.nih.gov/bioart provided by the NIH BioArt source were used in this figure.

Implemented as embedded 3D microstructures, µCodes were engineered to be directly incorporated into culture or sample collection surfaces and transition directly between brightfield, fluorescence, confocal, and scanning electron microscopy (SEM) techniques, as well as laser dissection and/or micromanipulation to enable targeted downstream molecular analyses (**Figure** **1**a). We designed the µCodes grid system to ensure at least one marker appears within the field of view of the most constrained (the bottleneck) instrument (Figure 1b). For our design, we considered the maximum field of view of an instrument to be the field of view in which corresponding magnification, the targets, here the cells, are clearly distinguishable. We determined element dimensions based on the resolution across all integrated instruments. Fields of view ranged from 1.3 × 1.3 mm (10X objective, fluorescence microscopy) to hundreds of microns (typical SEM) (Figure 1c). For our implementation, confocal microscopy represented the bottleneck instrument, with a 600 × 600 µm field of view at 20X magnification which necessitated a 500 µm inter‐marker spacing. This configuration yielded a marker density of 4 µCodes mm^−^^2^ or 400 µCodes cm^−^^2^. While the designs parameters reported here are optimized for our experimental conditions, µCodes can be tuned to accommodate a range marker densities and footprints (Figure 1d,e). Table S1 (Supporting Information) includes a comparison of µCodes features and footprints across various designs. To enable widespread dissemination, we open‐sourced the code that details the GDSII microfabrication masks for µCodes (Experimental Section).

### 2.2 Coordinate Systems and Coding Architectures of µCodes

To accommodate varying sample geometries and instrument‐specific constraints, we explored multiple coordinate and coding systems for µCodes implementation. While Cartesian coordinates provide intuitive readability and align with most commonly used standards in imaging instruments, alternative coordinates systems such as Polar and Parabolic could benefit applications where the sample has a specific dynamic, region of interest, or the codes need to match the instrument characteristics such as fisheye field of view. Our demonstrations primarily employ Cartesian coordinates, owing to their compatibility with conventional imaging platforms.

Numbers and references representing the coordinates can be represented in any base, and therefore each variation needs to be evaluated for compactness, information density, human readability, machine interpretability, and microfabrication fidelity prior to embedding them into the imaging platforms. Although base‐10 numerical digits are most intuitive for human interpretation, their complex curves and small lines challenge machine vision algorithms and complicate fabrication at microscale, the latter of which can cause loss of data or patterns during molding or pattern transfers in manufacturing. Considering the microfabrication processes constraints, given a critical feature dimension, we therefore developed several coding schemes using simple geometric elements e.g., rectangles, that maintain structural integrity during microfabrication and facilitate machine vision and automation of image processing.

**Figure 2:**
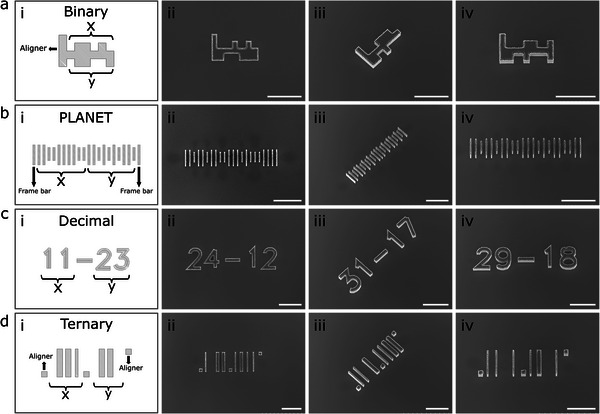
µCodes variations. ai) Binary µCodes design. Binary µCodes are comprised of an x vector, a y vector, and an align mark. The align mark shows the orientations of the devices and is used as a universal measurement point across the device. bi) Postal Alpha Numeric Encoding Technique (PLANET) µCodes design. PLANET design is mainly utilized in postal services, but could be useful in biomedical microdevices. The PLANET coding system is comprised of x and y vectors as well as two frame bars, acting as align marks and measurement points. ci) Decimal µCodes design. The decimal design is based on the Numerical digits. This coding system is the easiest to read for the human eye but, given the same minimum feature size, it occupies a larger footprint in comparison with other coding systems. di) Ternary µCodes designs. The Ternary design introduces thickness as a variable to the binary design. Same as the binary design, the ternary design is comprised of 2D and align marks for measurements. aii–aiv, bii–biv, cii–civ, dii–div) SEM images of fabricated µCodes of Binary, PLANET, Decimal, Ternary coding systems. Scale bars are 50 µm.

We implemented and tested five specific encoding schemes: Cartesian Binary (**Figure** **2**a), Cartesian PLANET (Postal Alpha Numeric Encoding Technique) (Figure 2b), Cartesian Decimal (Figure 2c), Cartesian Ternary (Figure 2d), and Polar Decimal (Figure S1, Supporting Information). Each µCodes system incorporates an embedded or separate reference feature that serves as an alignment mark and measurement origin. These reference features may take the form of pixels, bars, or structural distances and angles (Figure 2ai,bi,ci,di). Binary µCodes (Figure 2a) are comprised of x and y vectors with an align mark indicating the device orientation and measurement point and offer the simplest structure for human and machine readability while maintaining fabrication reliability. PLANET µCodes (Figure 2b) are composed of x and y vectors and two frame bars, acting as align marks and measurement points. Decimal µCodes (Figure 2c) are based on the Numerical Digits and are the most familiar to the human eye. Ternary µCodes (Figure 2d) design principles overlap with those of the binary but introduce thickness as a variable. Table S1 (Supporting Information) includes a comparison of the footprint and size of these variations. Binary µCodes are the most compact form of these structures and therefore we favored this design for further explorations.

Therefore, by embedding unique 3D geometric markers and a relational coordinate system directly into sample collection surfaces, we have developed a robust correlative microscopy tool to enable seamless transition between multiple imaging modalities, unambiguous target identification, and data integration.

### 2.3 Scalability and Recognition of µCodes

**Figure 3:**
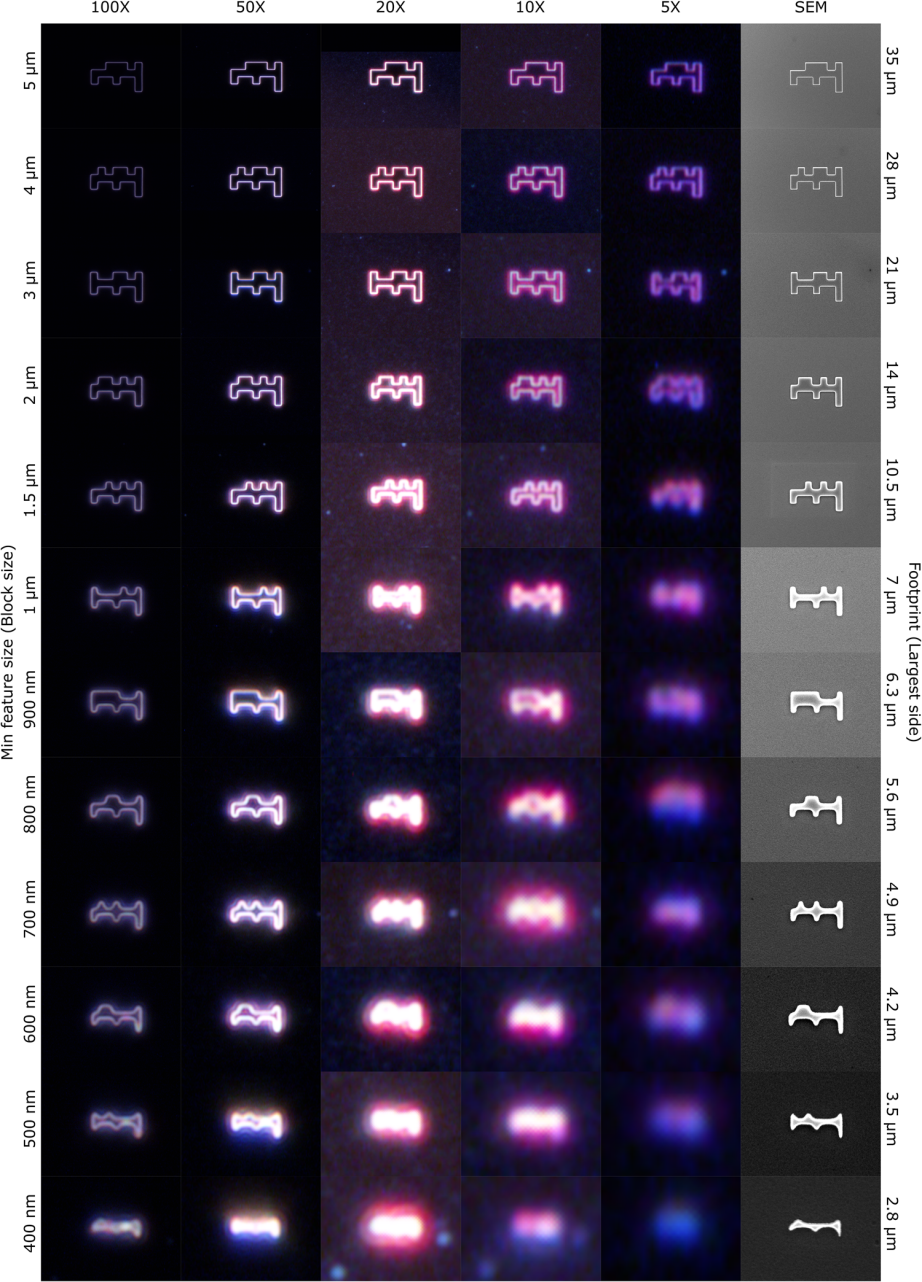
µCodes are scalable for specific applications. Dark‐field images from 100X, 50X, 20X, 10X, 5X objective lenses of an optical microscope along with SEM images of the same codes are illustrated. The recognition of the µCodes, is dependent on the fabrication quality, imaging conditions, and instruments, as well as the specific application of the platform. The code size could be selected such that the footprint of the µCodes, matches the field of view of the instruments in the pipeline of the intended application. The substrate in this figure was created by etching silicon for 600 nm using Bosch Process. Minimum feature size is the smallest feature size of the structure fabricated and matches the side size of each square bit making the µCode. Footprint is the length of the structure, which in this case equals seven times the minimum feature size (Due to the 6‐bit binary code + 1‐bit align mark). The first magnification on which the first 6 SEM images are taken (in Min feature size: magnification format) are: 5:700X, 4:700X, 3:700X, 2:1500X, 1.5:2200X, 1:2000X. If for the purpose of an example, we assume that the structures with a minimum feature size of 2 µm and larger are visible and distinguishable under 5X optical and the structures with a minimum feature size of 1 µm and larger are visible and distinguishable under 10X optical, this implies a 140‐, 140‐, 140‐, 300‐, 220‐, and 200‐fold magnification range in this specific application. While we designed the µCodes with a 250X dynamic range in mind, this example illustrates the applicability and scalability of the µCodes across a variety of ranges and shows the adaptability of the platform for the user's specific application.

We designed the µCode system with tunable code variants and scalable footprints that can be matched to the instrument field of view (FOV) and the scale of samples in a given application workflow. To demonstrate the scalability of the system, we fabricated µCoded substrates with feature sizes range from 400 nm to 3.5 µm by etching silicon to 600 nm depth using the Bosch process (**Figure** **3**). Identical µCode structures were then imaged across a wide range of magnifications using optical microscopy (5‐100X objectives) and SEM.

Our design targeted a 250X dynamic range to test compatibility across applications spanning low‐magnification optical microscopy to high‐resolution SEM. For example, structures with feature sizes of 1 and 2 µm that were resolvable at 10X and 5X magnification respectively, achieved dynamic ranges of 140–300X across the optical and SEM imaging conditions tested (Figure 3). In this instance, while µCodes with 400 nm feature sizes fabricated with this process were detectable with SEM, we found that 500 nm feature sizes were required for reliable detection with optical methods (Figure 3). Collectively, these data reveal the influence of magnification, imaging conditions, and fabrication precision on the limits of feature recognition. While additional optimization and/or different fabrication processes might increase detection limits, these data credential µCodes for submicron, multi‐modal applications.

### 2.4 Integration of µCodes in Cellular Characterization Devices

**Figure 4:**
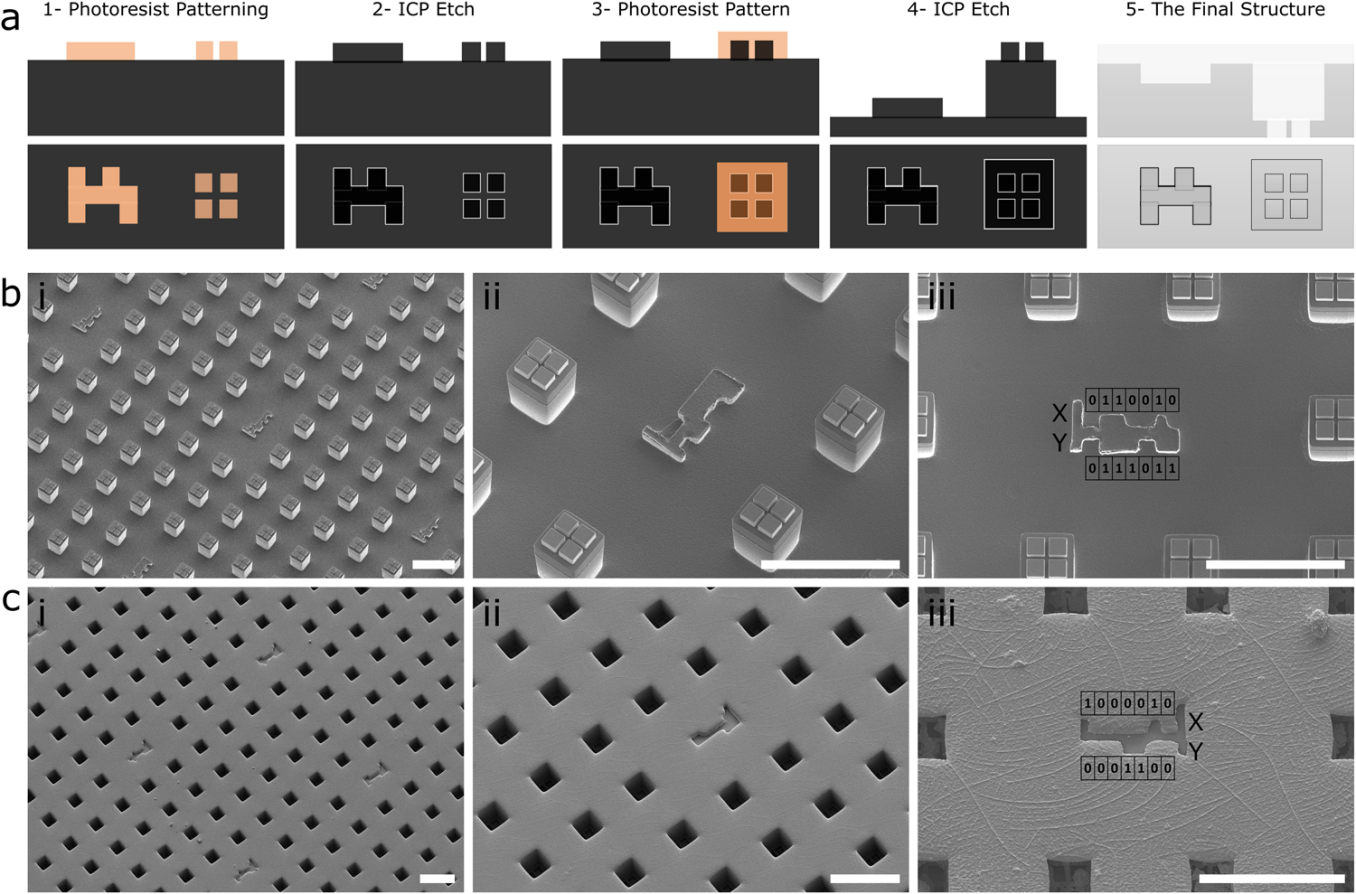
Microfabrication of µCoded filters for cell characterization. a) A proposed microfabrication flow for creating µCodes on mesh filters with a pattern transfer technique using 2‐step lithography and etch. The structure is fabricated on a silicon wafer as a mold. The structure is transferred to a resin to form the final device. bi–biii) Fabricated µCodes silicon structures. ci–ciii) Replica of the fabricated structures in plastic. Plastic devices are used for isolation, processing, and characterization of the target cells. biii, ciii) Illustrations of the bits in the µCodes that make up the x and y components of the codes. Scale bars are 100 µm.

To examine the practical utility of µCodes, we integrated them into microwell^[^ ^37^ ^]^ filter devices designed to study CTCs that are isolated from whole blood samples. We selected binary µCodes for this application due to their simple geometric structure that minimized data loss during feature transfer etching step of the fabrication process and to optimize readability at small feature sizes. We fabricated filters with 11 µm pore size using a novel pattern transfer technique (described in Experimental Section) that positioned the µCodes structures at a lower height on the silicon wafer (**Figure** **4**a) and allowed high‐fidelity replication of µCode features. This approach enabled the transfer of µCodes markers from the silicon master mold (Figure 4b) to plastic substrates (Figure 4c) to form final “µCoded‐Filters.”

µCodes that were integrated into µCoded‐filters were fabricated such that they appeared at a different focal plane relative to the cell trapping structures (Figure 4cii). While this required collecting images at multiple focal planes (i.e., z‐stack), this strategic positioning preventing µCodes from interfering with cellular imaging (Figure S2, Supporting Information). Computational fluid dynamics simulations (Comsol) confirmed that µCodes did not significantly affect the flow distribution and shear field of the filters, indicating the preservation of device functionality (Figure S3, Supporting Information).

While µCoded‐filters were patterned into polymers (Figure 4c), we emphasize that they and the broader µCode system are agnostic to substrate material. We demonstrate successful application of µCodes in silicon (Figure 2), glass (Figure S1a, Supporting Information), metals (Figure S1a, Supporting Information), and suggest that other biocompatible surfaces would be receptive to accepting µCodes using standard microfabrication methods such as photolithography, etching, deposition, or molding processes. Thus, the versatility of µCodes ensures that they can be integrated into virtually any sample preparation platform while maintaining their geometric integrity and encoded information. To enable widespread dissemination, we open‐sourced fabrication masks for several µCoded‐filter variants (Experimental Section).

### 2.5 Multimodal Microscopy Navigation and Target Tracking by µCodes

Standard biological workflows typically involve multiple sequential steps: sample isolation, staining, multimodal imaging, and downstream molecular analyses such as genetic sequencing, mass spectroscopy, or electron microscopy (Figure 1a). For example, when processing a whole blood sample to study circulating tumor cells (CTCs), investigators must isolate, perform immunostaining, image sequentially at low‐ and high‐resolution to optically identify and study targets of interest, then complete downstream analysis. For each step in the workflow (Figure 1a) it is imperative to follow the target(s) of interest to ensure data is collected from the intended target(s). µCodes provide consistent spatial referencing throughout these processes to ensure seamless transitions between imaging steps and that the same cells or regions of interest can be reliably relocated, and target data can be integrated.

**Figure 5:**
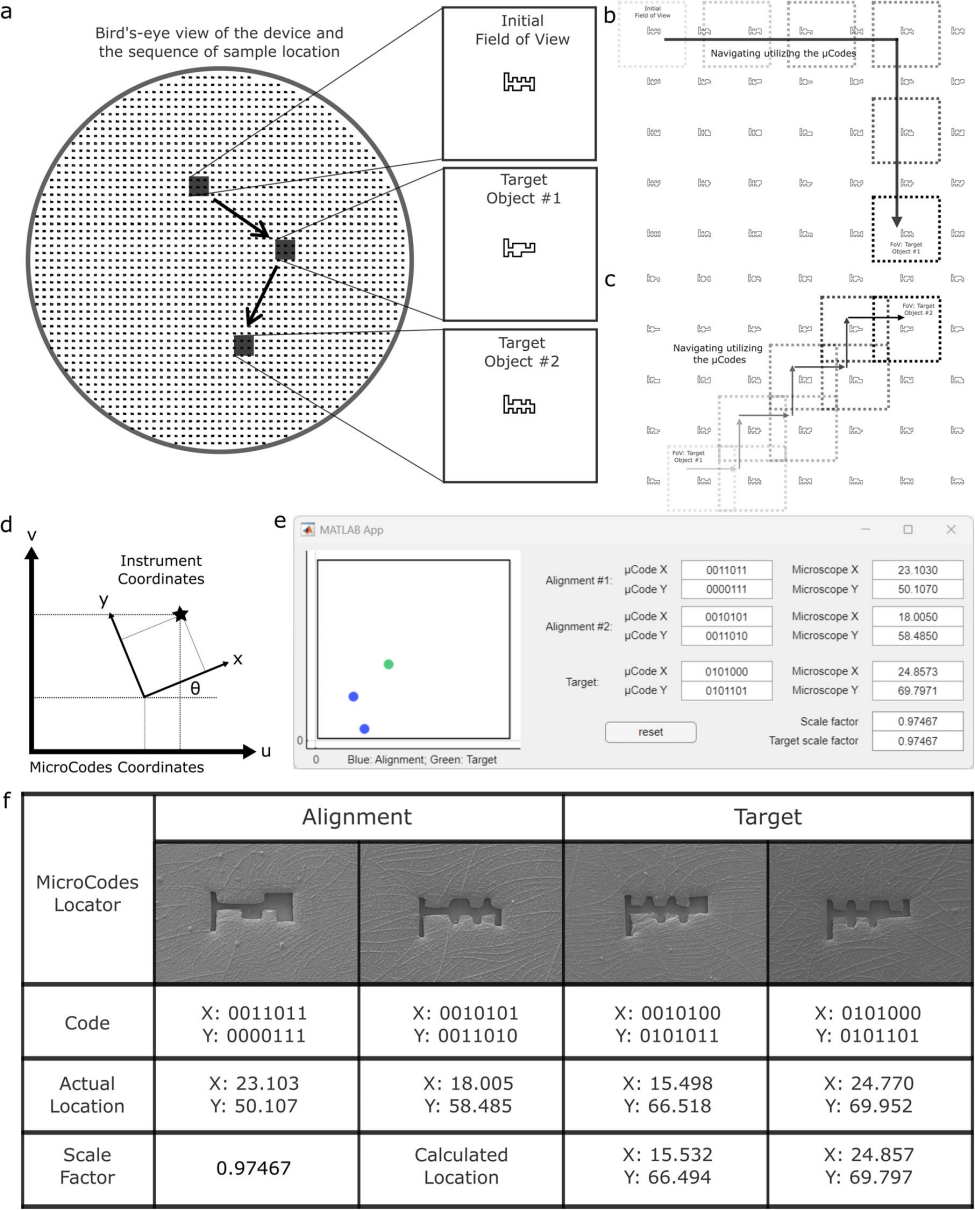
µCodes navigation and µCodes locator app: a) An illustration of the Bird's eye view of the device. After an initial imaging and identification of the targets, e.g., via fluorescent imaging, the µCodes are utilized for navigation over the device to the location of the target, e.g., in a confocal or electron microscope. b) An example of sequential navigation, by following the µCodes first in x direction, then in the y direction to reach the target. The square represents the field of view (FoV) of the instrument. c) An example of sample navigation by alternatingly following the µCodes in x and y directions. d) An illustration of mapping the µCodes coordinates to the coordinates of an instrument both using Cartesian coordinates. e) µCodes locator MATLAB app. To use the software, the user first pinpoints two random µCodes and enters them along with their instrument coordinates into the application. The app then calculates the relations between the two coordinates. The app can then calculate the instrument location for any given µCode. f) An example of navigation using µCodes in an SEM. The target coordinates are calculated using the app for the given µCodes. As seen in the figure, the calculated location is not exact, which is caused by the non‐uniform changes in dimensions of the device due to chemical processes and water absorption during the sample preparation steps. The scale factor is the metric that shows this deformation among the first two alignment µCodes.

Users can navigate samples manually using the µCodes grid (**Figure** **5**a). This functionality proves particularly valuable in multi‐modal imaging, where targets mapped to a particular µCode via one method (e.g., fluorescence microscopy) must be relocated in another (e.g., confocal or electron microscopy). Target relocation can be accomplished through sequential navigation along grid coordinates (Figure 5b) or through alternating movements in x and y directions (Figure 5c).

To automate navigation, we developed a MATLAB application that establishes the coordinate relationship between the instrument positioning system and the µCodes reference frame (Figure 5e). The software requires users to locate just two arbitrary µCodes and input their coordinates, from which it calculates transformation parameters and substrate scaling factors (Figure 5d). The application then guides users to the desired targets by calculating their locations (Figure 5f), accommodating non‐uniform substrate deformations that may occur during sample preparation. The mathematical relations behind the coordinate mapping are described in the supporting information. Initially developed in MATLAB, we provide additional implementations in C++ and Python (Experimental Section).

µCodes provide consistent spatial referencing across multi‐step biological workflows, enabling reliable target tracking and relocation between different imaging modalities. This system allows for both manual navigation along grid coordinates and automated target location through the instrument software integration or an independent application that requires minimal user input to establish coordinate relationships.

### 2.6 µCoded‐Filters Facilitate Correlative Microscopy Study of Cancer Cells

We tested µCoded‐Filters’ capacity to enable correlative microscopy study of the populations of cancer cells. We captured HEY‐A8 cancer cells that stably express GFP directly using our coded filter system. Cells were fixed, stained with DAPI, and imaged without further modification. Using lower‐resolution (20X) confocal microscopy, we collected fluorescent images of HEY‐A8 captured with the µCoded‐Filters. We simultaneously collected transmitted light to detect the associated µCodes.

We then picked regions of interest containing dozens of HEY‐A8 and several µCoded markers to initially validate cross‐image modality sample identification. The same µCoded‐Filter was then processed for SEM imaging (described in Experimental Section). We detected ≈73% of cells in the same position as they were in optical images, ≈14% moved (defined as <10 µm from original position), while ≈13% were lost (Table S2, Supporting Information). We note that loss is likely due to the harsh chemical treatment and multiple washing steps required of the SEM sample preparation process and not inability to re‐identify µCodes. During SEM imaging, the unique information encoded in individual µCodes and their relational coordinate system facilitated precise and rapid navigation to the region of interest and sample re‐identification. µCodes dependent navigation is even robust to rotations or shifts in imaging reference frame, commonly observed with multimodal approaches (Figure 5a).

**Figure 6:**
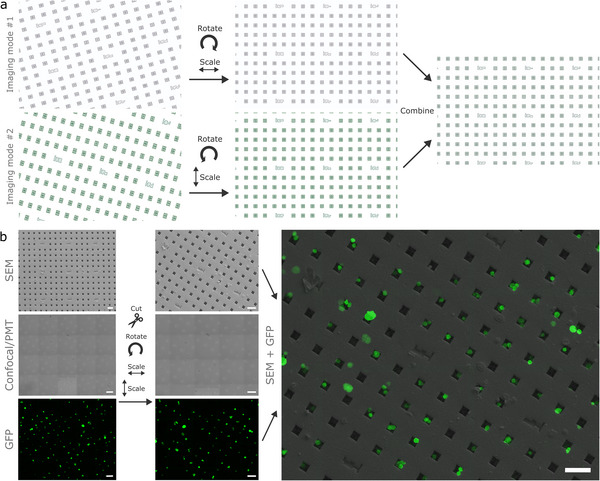
µCodes image alignment and overlay. a) Theoretical illustration of alignment and fitting of images from different imaging systems (e.g., confocal microscope, SEM, TEM, surface profiler) or imaging mode (e.g., different magnification, detector, camera). In this theoretical example, the images undergo rotation and scaling transformation. b) Exemplary showcase of image fitting for Scanning Electron Microscopy (SEM) and Confocal images of HEY A8 cells. The T‐PMT(transmitted light) and SEM images are used for setting alignment parameters. Not all cells are in the same position in the SEM image due to the loss of the cells during the chemical processing, which accounts for some mismatch in the Green Fluorescent Protein (GFP) and SEM images. Scale bars are 50 µm.

**Figure 7:**
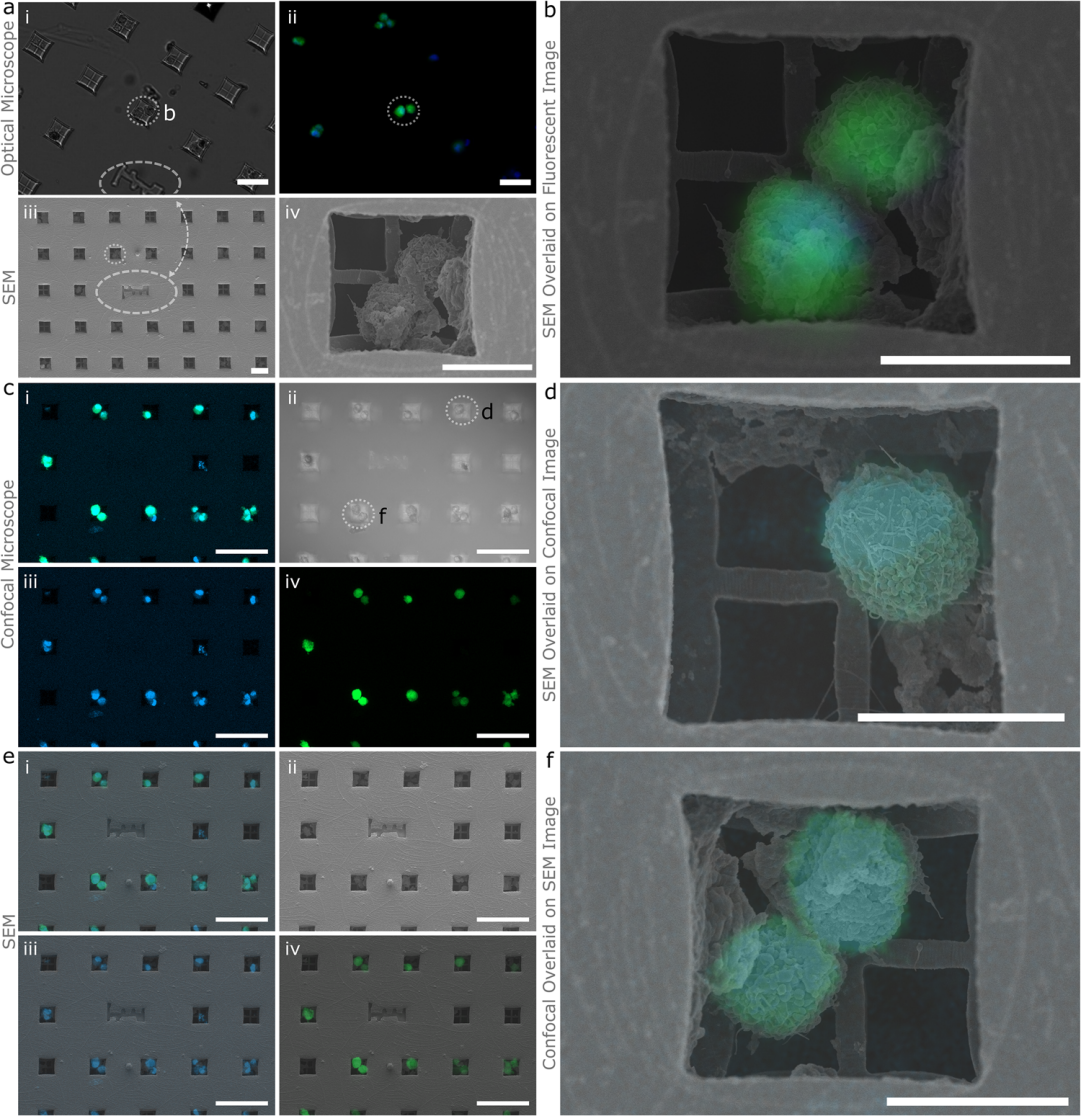
µCodes for co‐registration of images and targets: ai, aii) Brightfield and fluorescent images of the target of interest identified during the initial imaging. aiii) µCodes are utilized to locate the same area of interest and aiv) cells of interest under Scanning Electron Microscope (SEM). b) An overlay of fluorescent on SEM image of HEY A8 cells. c) Confocal microscope image of HEY A8 cells. ci) GFP + DAPI, cii) T‐PMT(transmitted light), ciii) DAPI, and civ) GFP. e) SEM image of the same region located and aligned with µCodes. ei) SEM + GFP + DAPI, eii) SEM, eiii) SEM + DAPI, eiv) SEM + GFP. d) SEM image overlaid on confocal microscope image of a Hey A8 cell. f) Confocal microscope image overlaid on SEM image of HEY A8 cells. All alignment parameters are fitted for SEM and PMT images. Other channels are scaled and transformed using the same parameters as the PMT. Scale bars in (ai–aiii) are 50 µm, aiv) 20 µm, c,e) 100 µm, and b,d,f) are 20 µm.

µCodes were then used to co‐register multimodal images (**Figure** **6**a). Co‐registering data from different imaging systems or modes involves applying geometric transformations to align and correct distortions that may occur between different imaging modalities (Figure 6b). µCodes’ foundation in geometric features is ideally suited for this task. We then explored applications for µCoded‐Filters to enable correlative microscopy at the single‐cell, high‐resolution level. After an initial imaging screen at low (10X) resolution where we identified particular cells of interest, we then follow these exact cells through all subsequent multimodal imaging steps utilizing µCodes to guide navigation and cell identification through high resolution (20X, 63X) confocal microscopy and SEM (**Figure** **7**a,c,e). Embedded µCodes information was then used to align, correct aberrations, and ultimately synthesize a high‐resolution cell‐level fluorescent‐SEM dataset (Figure 7b,d,f).

These results demonstrate that µCodes were successfully integrated into existing correlative microscopy processes. µCodes facilitated precise navigation and identification of regions of interest across different imaging platforms. µCodes’ 3D geometric features provided a robust spatial reference frame that remained invariant to spatial distortions induced by different imaging systems. Both features then allowed for accurate co‐registration and synthesis of high‐resolution fluorescent and SEM datasets at the single‐cell level.

### 2.7 Cell and Tissue Culture Applications for µCodes

**Figure 8:**
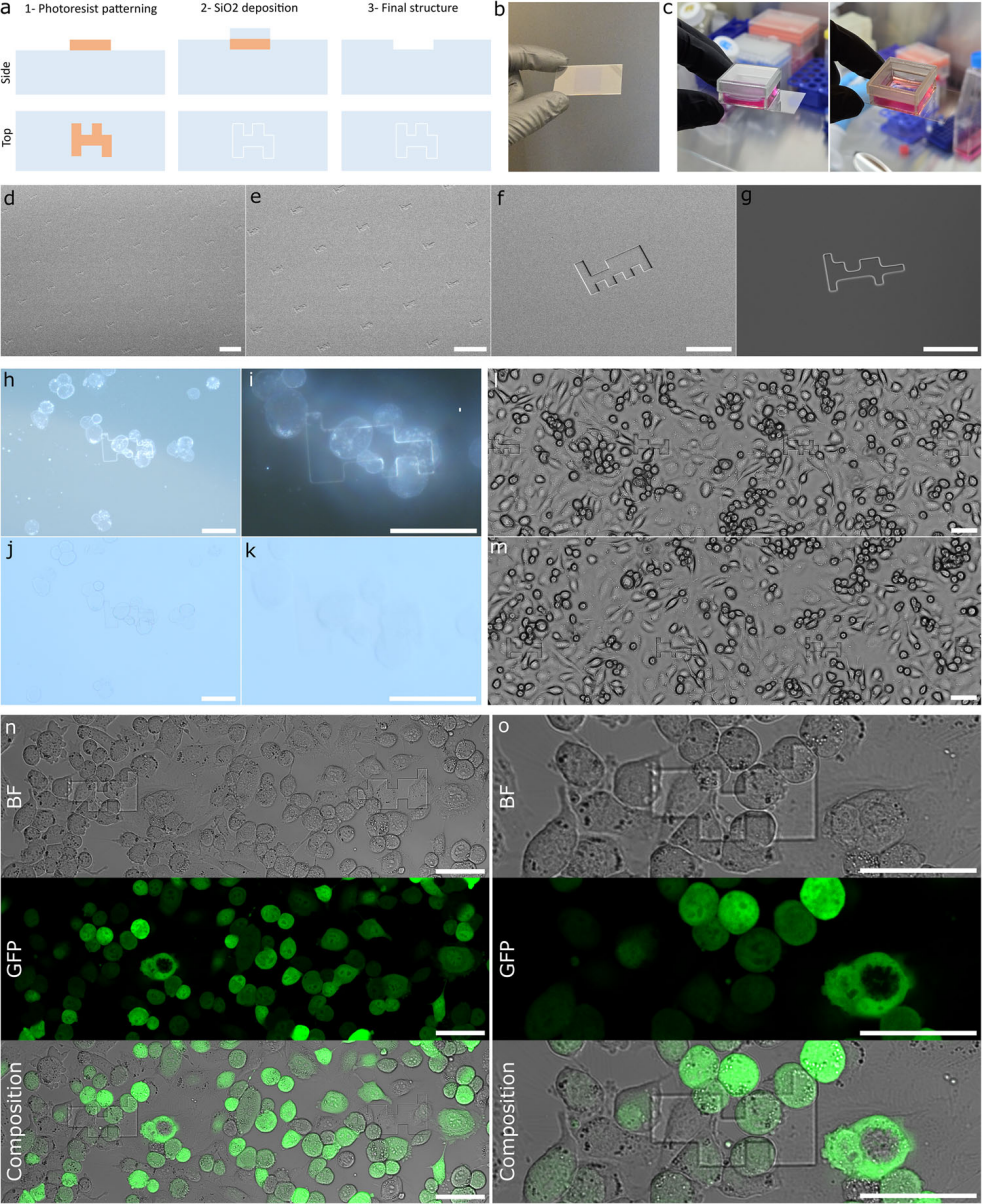
µCoded slides and dishes for cell culture: a) Microfabrication flow for creating µCodes on glass slides using a conventional lift‐off technique. The structure is fabricated on a standard glass slide (either an imaging slide or a coverslip), with SiO_2_ deposited on the slide to create the µCodes structures. b) A sample µCoded slide. c) A sample µCoded culture dish with glass cover on top. d,e) Low Magnification mode (LM), f) Secondary Electron (SE), and g) Low Angle Backscattered Electron (LA‐BSE) SEM images of the µCoded slide. h,i) Dark Field (DF) and j,k) Bright Field (BF) images of a cell‐plated µCoded slide 1‐h after plating cells at room temperature. Images i,k) were taken with a 50X objective while h,j) with a 20X objective. Comparison of DF and BF images shows that µCodes are distinguishable when covered with cells under 20X objective but only distinguishable in DF imaging under 50X objective. l,m) are BF images of ID‐8 cells 24 h after being plated on µCoded slides and cultured. n,o) Bright Field (BF), GFP, and composite confocal microscope images of ID‐8 cells 24 h after plating and growth in an incubator. o) is a zoomed‐in image of the same µCode as (n). The µCodes remain visible and distinguishable even when covered with cells. The µCodes structures are not visible on GFP channel, which implies µCodes do not interference with fluorescent imaging. This data suggests that the µCoded slides and dishes are suitable for cell culture applications. The visibility and distinguishability of the µCodes depend on the use‐case, imaging instrument, and the material used for fabrication. The µCodes structures shown were fabricated using 150 nm SiO_2_ deposition, but visibility and applicability can be customized for specific applications by modifying structural dimensions (e.g., 2‐micron depth) or materials (e.g., ITO). Movie S1 (Supporting Information) illustrates an image series cycling through the Z‐stack of (n). Scale bars in (d,e) are 200 µm and (f–o) are 50 µm.

To evaluate the suitability of µCodes for live‐cell imaging and culture applications, we fabricated µCoded slides by depositing a 150 nm SiO_2_ layer using a standard lift‐off process on #1.5 glass coverslips (170 µm thickness), a widely used substrate for high‐resolution optical imaging (**Figure** **8**a). These µCoded coverslips were compatible with both standalone imaging (Figure 8b) and as the base of custom culture chambers (Figure 8c). SEM imaging confirmed the fidelity of the µCode structures across the substrate surface (Figure 8d–g).

We next assessed the performance of µCoded slides during live‐cell imaging. µCoded slides were either gas (ethylene oxide) or autoclave sterilized prior to plating ID8 ovarian epithelial cells that stably express the green fluorescent protein (GFP) (Experimental Section). At 20X (Olympus MX61), µCodes remained visible under both brightfield (BF) and darkfield (DF) illumination, with DF providing superior contrast (Figure 8h,j). At 50X, µCodes were distinguishable under DF (Figure 8i) but were partially obscured in BF by overlying cells (Figure 8k). We note that floating cells produced the greatest occlusion of µCode patterns whereas adherent monolayers did not (Figure 8l,m).

To assess performance across platforms, we imaged the same samples using a low‐cost Echo Rebel microscope, commonly used for viability and maintenance checks, and a high‐end Olympus FV4000 system (Figure 8l–o). In both cases, µCodes remained visible beneath the confluent cells. Additionally, we detected no autofluorescence from µCodes (Figure 8n,o), confirming results from µCoded filters.

**Figure 9:**
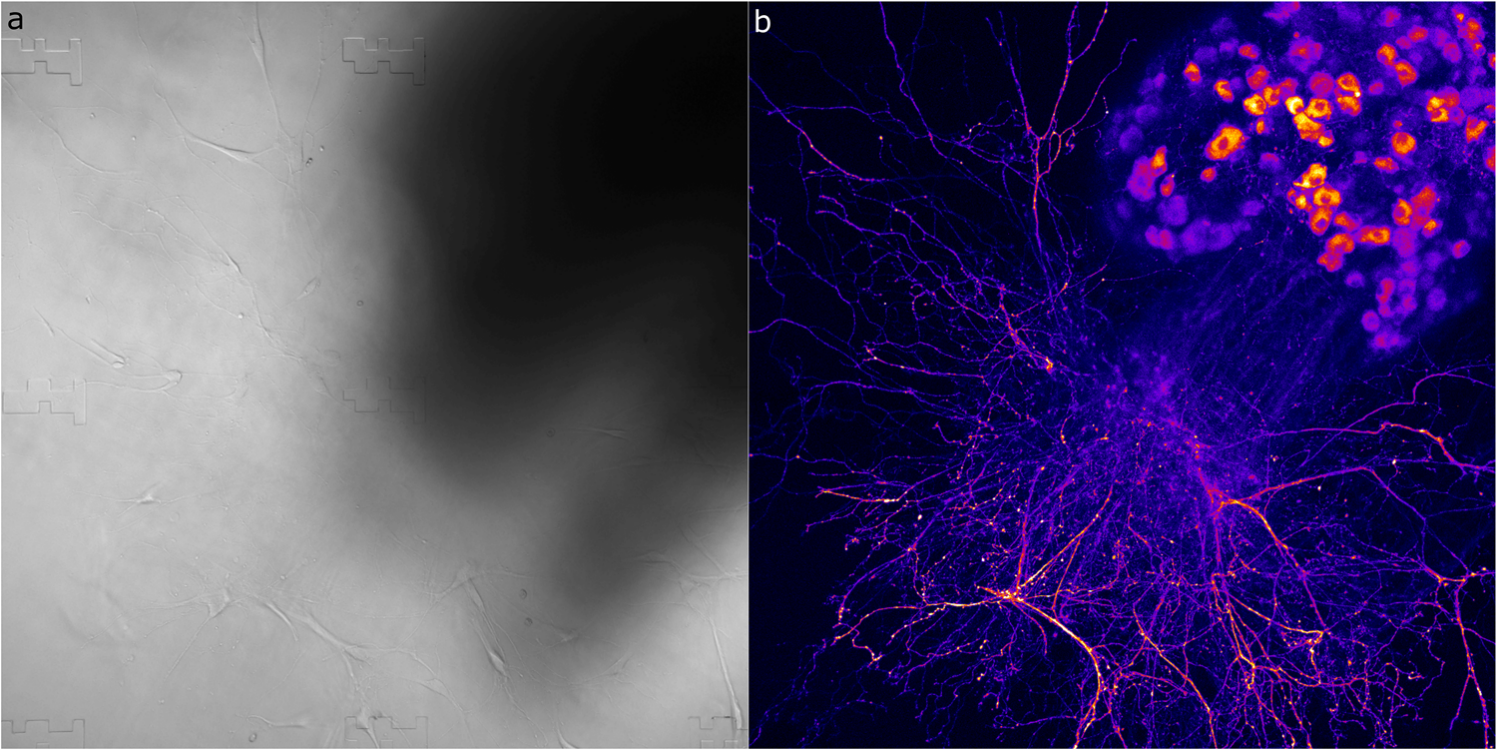
µCodes enable referencing in tissue culture. a) Brightfield (BF), b) maximum intensity projection (pseudocolored fire LUT) of tdTomato expression of primary cultured dorsal root ganglion (DRG) neurons organ explant. DRGs were extracted from *Actl6b^Cre^:Ai14* mice and plated on µCoded slides for culture. Imaging was conducted 72 h after plating. This figure illustrates the applicability of µCodes in tissue culture. a) is a Z plane where the µCodes are in focus. b) is Z project with maximum pixel intensity. Movie S2 (Supporting Information) illustrates an image series cycling through the Z‐stack of this figure.

To extend the platform to 3D, organoid, and tissue models, we utilized dorsal root ganglion (DRG) explants from Actl6bCre:Ai14 mice, a pan‐neuronal reporter line in which neurons constitutively express the tdTomato fluorescent protein (**Figure** **9**). µCoded slides were coated with a layer of Matrigel, and DRGs were plated on top and covered with media before incubation at 37 °C and 5% CO_2_ (Experimental Section). 72 h after platting, imaging (20x confocal) revealed the µCode focal plane was easily separated from the fluorescently labeled cells enabling independent visualization of both reference µCodes and biological specimens (Movies S1 and S2, Supporting Information).

These results demonstrate successful integration of µCodes into cell and tissue culture workflows. µCode structures did not interfere with cell plating, adherence, or viability, and are composed of materials compatible with standard sterilization protocols. We note that µCode visibility depends on structural parameters, material properties, and imaging conditions. While surface‐based techniques like SEM cannot access buried codes, the platform remains valuable for transmission‐based imaging and longitudinal studies tracking cellular dynamics. To facilitate their dissemination, we open‐sourced the code and files to fabricate µCoded slides and imaging chambers (Experimental Section).

## 3 Discussion

Here, we introduce µCodes as an approach to enable robust navigation, mapping, and data integration for multimodal imaging through the incorporation of a microscale 3D engineered grid system. Our approach overcomes three key challenges of multimodal imaging and correlative microscopy. First, it provides an unambiguous sample reference frame capable of identification and tracking precise sample locations. Second, it is adaptable and cross‐modality compatible, enabling applications in optical‐, confocal‐, and electron‐microscopy. Third, it encodes a geometric coordinate system that aids sample navigation and does not interfere with primary data collection. Notably, µCodes can be easily incorporated into existing correlative microscopy workflows to provide a standardized quality control step.

**Figure 10:**
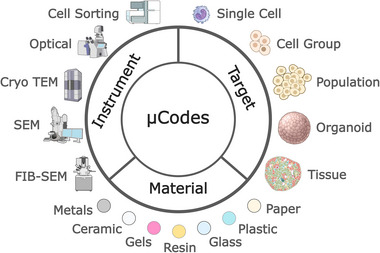
µCodes Canvas: µCodes are instrument, material, and target agnostic. µCodes could be embedded into metals, ceramics, gels, resins, glass, plastic, or paper. µCodes could also be utilized to assist in characterization of a variety of biological, such as single cells, cell populations, organoids, or tissues, or non‐biological samples. µCodes could be utilized to navigate, locate, map, or reference targets in a variety of instruments, such as optical microscopes, cell sorters, SEM, TEM, FIB, and surface profilers. Images available at https://bioart.niaid.nih.gov/bioart provided by the NIH BioArt source were used in this figure.

In addition to overcoming three key challenges of correlative microscopy, we highlight three additional benefits of our µCodes system (**Figure** **10**). First, µCodes are agnostic to substrate material. While we successfully demonstrate microfabrication and implementation of µCodes in silicon, plastic, and glass, their application can be extended to other substrate materials such as ceramics, paper, gels, or metals that may be required for specific correlative microscopy applications. Second, the geometric, 3D nature of µCodes means their encoded information can be readily detected with optical‐ and electron‐based microscopes, thereby imparting instrument agnostic capabilities. While not demonstrated here, µCodes’ performance capabilities would likely extend to surface profiling methodologies such as atomic force microscopy (AFM). Finally, we emphasize that µCodes systems could be readily applied to a variety of samples of interest beyond the cultured cells demonstrated here. For example, correlative microscopy or multimodal imaging studies of tissues specimens with complex and/or heterogeneous morphology would benefit from µCodes’ out‐of‐sample reference frame. This is particularly advantageous when attempting to co‐register data of fluorescent and imaging mass spectrometry when most current fiducial systems are single modality. While in this study we relied on human vision for identification to illustrate the applications and potentials of the µCodes, future work will include coupling the platform with machine vision and artificial intelligence models to create a user‐friendly applications pipeline and streamline the multi‐modal, multi‐instrument, or time‐series processes.

In this work, we use microfabrication techniques to create µCodes grids with individual feature sizes of 10 µm, comparable to a small cell size, and whole code sizes of ≈80 µm and demonstrate that this provides sufficient resolution for identification, navigation, and image co‐registration at the single‐cell level for optical, confocal, and SEM correlative microscopy studies. For even finer, potentially subcellular correlative microscopy needs, feature sizes can be scaled down to ≈1–5 µm to closely match the smaller observation window of the intended application. Demonstrated here, µCodes can be scaled down to less than 1 µm pixel size without fundamentally changing the microfabrication process. The theoretical limits for individual feature sizes of µCodes would approach the tens of nanometer scale used in manufacturing of microprocessors. While process optimization and material selection would be critical, such small‐scale applications may have utility in the study of biomolecular condensates or single‐molecule tracking.^[^ ^38^ ^]^

As was recently demonstrated through the development of GelMap^[^ ^19^ ^]^ technology, the ability to intrinsically calibrate biological samples and correct for experimental deformation induced either intentionally, as is the case with expansion microscopy or unintentional as what is often the result of different environmental factors during data collection, is enabling for microscopy. As µCodes necessarily occupy 3D space (a serendipitous byproduct of the microfabrication process) and if they are patterned in resin or other compliant substrates, as we have demonstrated here, µCodes would provide a similar, albeit 3D, calibration curve to correct for local anisotropies in expansion and contraction due to changing environmental conditions that occur between optical and electron microscopy. We do acknowledge that this depends on deformations translating uniformly from sample to substrate or vice versa and that sample‐substrate interactions remain consistent. Figure S4 (Supporting Information) illustrates how local µCodes could be utilized for local isotropic and anisotropic deformation compensation. The implementations demonstrated here have heights that range from 150 nm to 6 µm which limits the extent to which they can calibrate z‐dimensional changes but updates to the design and/or microfabrication processes that increase the z‐dimensional feature size may expand their utility. µCodes foundation in 3D geometries imprinted into the sample substrate also liberates their dependence on fluorescence signals. Fluorescence independent information not only enables widespread and rapid adoption in EM applications where fluorescent detectors are typically absent from legacy and the majority of contemporary instruments,^[^ ^22^, ^39^ ^]^ it frees up a fluorescent channel to expand multiplexed immunohistochemical or genetic labeling studies.

Another key advantage of µCodes is their ability to reduce alignment errors between imaging modalities. This is particularly beneficial for both the large field of view and high‐resolution imaging studies. By providing a physical reference for image alignment, µCodes improve the reliability of correlative imaging techniques, streamlining sample identification, instrument navigation, and data integration workflows. In the future, µCodes can be integrated into multimodal studies that incorporate downstream molecular analyses. For example, µCodes can guide the postimaging collection of single cells for downstream omic‐type studies by validating cell identity and confirming collection into a micropipette. Figure S5 (Supporting Information) illustrates how µCodes could be utilized to guide sample collection for downstream analysis. We find this set of applications particularly relevant for integrated study of rare cellular events with clinical and scientific relevance such as circulating tumor cells.^[^ ^40^, ^41^ ^]^

More broadly, µCodes can be integrated into a variety of clinical imaging devices or workflows. For example, in the clinical setting, µCoded slides could direct diagnosticians utilizing multiple imaging platforms to specific regions of interest by referencing the grid coordinates, streamlining secondary imaging requests and consultation processes. We acknowledge that comfort with incumbent devices, end‐user education, and ease of incorporation into existing workflows (particularly as it relates to electronic medical records in the clinical setting) in addition to costs are all barriers to adoption. By open‐sourcing the technology, fabrication processes, and providing a diverse library of credentialed use cases, as well as µCodes capability to integrate into existing platforms and workflows, we hope to circumvent many of these obstacles to adoption in the academic, clinical, and industrial domains.

In summary, µCodes provides a simple and robust system to identify, map, and navigate diverse samples in correlative microscopy studies and to ultimately integrate multimodal data. We anticipate that this will aid the development and validation of multimodal imaging protocols and facilitate the widespread implementation of correlative microscopy into imaging applications that require robust and reliable calibration, both in basic science and in clinical applications.

## 4 Experimental Section

### Creation of the Masks

The structures and the subsequent masks for fabrication which include the grids, µCodes and the other microstructures were generated by a Matlab(R2023a) code. The structures were then directly written to a GDS file using the gdsii‐toolbox.^[^ ^42^ ^]^ The design unit size was 1 nm and the minimum feature was 400 nm. The structures were inspected using Klayout to ensure the correctness of the design. The following GitHub repository contains sample fabrication masks and codes for systematically producing GDSII microfabrication masks for µCoded structures: github.com/Arefvp/MicroCodes.

### Microfabrication of the Coded Filters

The fabrication flow for coded‐filters is illustrated in Figure 4a. First, a silicon wafer was washed using Acetone and IPA and rinsed with DI water. The wafer was coated with MICROPOSIT S1813 photoresist and exposed using Heidelberg Instruments MLA150. The sample was developed, hard baked at 100°C for 5 min, and etched 6 microns using Bosch process. The resist was then striped and a new layer of MEGAPOSIT SPR 220 was applied to the wafer. This layer was then exposed using Heidelberg Instruments MLA150 and developed using MICROPOSIT MF 319 Developer. The final resist structure covers the filter features but not the codes. Then the wafer was etched 40 microns using STS HRM ICP. This step results in the µCodes structures to be transferred down while the filter structures were protected. After etching, the wafer was stripped of the resist, washed with acetone/IPA and dried using nitrogen. A layer of (TRIDECAFLUORO‐1,1,2,2‐TETRAHYDROOCTYL) TRICHLOROSILANE (Gelest) was applied to the wafer and the structure was then cast to a PDMS mold. The mold was used to make resin (either FormLabs Surgical Guide Resin, BioMed Amber Resin, or MD700) replicates of the structure.

### Microfabrication and Imaging of the Size Sweep Structures

To fabricate the structures illustrated in Figure 3, a silicon wafer was coated with MICROPOSIT S1805 photoresist and exposed using Heidelberg Instruments MLA150. The wafer was etched 600 nanometers with Bosch process using STS Pegasus ICP. The resulting structures were imaged using an Olympus MX61 optical microscope and objective lenses of 100X, 50X, 20X, 10X, and 5X under darkfield settings of the microscope. The SEM images were taken using a Hitachi SU8230 FE‐SEM.

### Fabrication of Culture Slides and Dishes

The fabrication process is illustrated in Figure 8a. The µCode structures were fabricated using standard lift‐off process. After photolithography using MICROPOSIT S1813 photoresist exposed with a Heidelberg Instruments MLA150, 150 nm of SiO2 was deposited using Denton Explorer E‐Beam Evaporator, on a standard #1.5 glass coverslip (170 µm thickness). #1.5 glass coverslips were chosen since they were commonly used for high‐resolution biological sample imaging. The slides were either sterilized directly for use in cell or tissue culture or were mounted with a chamber and a lid to make a culture dish similar to commercially available chambered coverslips for cell culture.

The chamber and cover lid parts were designed in SolidWorks, exported to STL files and printed on a FormLabs Form 4B 3D printer using BioMed Clear resin. After the post‐processing following the resin manufacturer protocol, a small amount of the same resin was applied to the surface of the chamber surface facing the glass. The glass slide was then pushed against the chamber part and exposed to UV to cure. The same process was done for placing a 22 mm by 22 mm glass slide on the cap part. Both the glass slides and the chambers were sterilized either using autoclave or Ethylene Oxide (EtO).

The following GitHub repository contains GDS microfabrication mask for fabricating the µCoded slides, and STL files for 3D printing the chamber and the lid: https://github.com/Arefvp/MicroCodes/tree/main/CultureSlides.

### Cell Culture and Immunofluorescent Staining

Stably GFP‐expressing HEY‐A8 cells were maintained in RPMI 1640 (Mediatech) supplemented with 10% FBS (Fetal Bovine Serum; Atlanta Biologicals) and 1% antibiotic‐antimycotic solution (Mediatech). Media were supplemented with Geneticin G418 sulphate (GIBCO) and Puromycin (GIBCO) selective agents to maintain reporter expression. Adherent monolayer cultures were maintained at 37 °C in 5% CO2. Cells were collected by centrifugation after trypsinization (GIBCO 0.25%). Cells were resuspended in PBS and isolated using the fabricated filter microstructures (µCoded‐Filters). Cells were then fixed directly on µCoded‐Filters with 4% paraformaldehyde (PFA) for 10 min at room temperature (RT) and washed with 4 mL of PBS. Cells were incubated in a 1:1000 dilution of DAPI for 10 min at RT and washed with 4 mL of PBS.

Stably GFP‐expressing ID8 cells were maintained in DMEM‐GlutaMAX™ (GIBCO) supplemented with 10% FBS (Fetal Bovine Serum; Atlanta Biologicals) and 1% antibiotic‐antimycotic solution (Mediatech). Media were further supplemented with Puromycin (GIBCO) selective agents to maintain reporter expression.

### Mice

All mice were housed, and all experimental procedures were carried out in the Department of Animal Resources (DAR) at the Georgia Institute of Technology (GT). All animal procedures were approved by the Institutional Animal Care and Use Committee at GT. Animals were maintained under constant environmental conditions (temperature in rooms is 68–72 °F and humidity 30–70%), with food and water provided ad libitum in a 12/12 h light/dark cycle. Adult mice from strains *Actl6b^Cre^* (no. JAX  027826) and Rosa‐lxl‐tdTomato (Ai14, no. JAX 007914) were purchased from the Jackson Laboratory. Experimental animals were generated by crossing *Actl6b^Cre^* line with the tdTomato reporter line to label neurons. All cre lines were maintained in a B6 background and were viable and fertile with no detectable abnormal phenotypes.

### Tissue Harvesting

Dorsal root ganglia (DRG) were harvested as previously described with slight modifications.^[^ ^43^ ^]^ Briefly, mice received a lethal dose of isoflurane anesthesia (5%) until respiration rates dropped below 10 beats min^−1^. Then, the animals were transcardially perfused with a vascular rinse containing heparin to remove blood. The spinal column was then extracted, cleaned of muscle and connective tissues. Ventral bilateral cuts were made from T2 to L5 spinal levels exposing the ventral aspect of the spinal cord and bilateral roots in a sterile fashion. DRG were dissected under magnification, taking care to remove proximal and distal aspects of roots to minimize supporting cell contamination during cultures. After extraction, DRG were placed in ice‐cold HBSS until plating.

### Dorsal Root Ganglia Plating

Sterilized (ethylene oxide and autoclave) µCoded slides were coated with 4uL drops (*n* = 10/slide) of Matrigel, which was allowed to stand at room temperature for 20–30 s. After partial solidification, DRG were removed from HBSS and inserted into the Matrigel drop. Plated DRG were then incubated at 37 °C and 5% CO_2_ for 5 min to encourage full solidification of the Matrigel. The slides were then placed in a sterile cell culture dish before being topped off with complete culture media to cover each Matrigel drop and incubated at 37 °C and 5% CO_2_ for 72 h before imaging.

### Fluorescent, Confocal, and Optical Microscopy

For experiments involving µCoded‐Filters illustrated in Figure 6, 7, the devices were transferred to No. 1 (0.14 mm) cover slips for imaging. Fluorescent microscopy was performed using an Olympus fluorescent microscope. Cover slipped µCoded‐Filters were then transferred to the Zeiss 900 laser scanning confocal microscope. Tile scans of images of large areas were acquired with a 20X objective (N.A. = 0.8). Z stacks of sections or high‐resolution cell imaging were acquired with a 63X objective (N.A. = 1.4) at a step size of 0.5 µm. Laser strength, gain, and other critical image acquisition parameters were held constant when imaging across samples. Stacks of images were processed and analyzed using Imaris (Bitplane) or imageJ (NIH) imaging software. Figures present images as either flat maximal projections of the sum of the Z‐axis optical slices or 3D rendering of Z stacking of 2D scanning images from different depths. Cell images in Figure 8h–k were captured under either Dark Field (DF) or Bright Field (BF) on Olympus MX61 optical microscope and objective lenses 20X and 50X as described in the Figure 8 caption. Images in Figure 8l,m were captured on an ECHO Rebel tissue culture brightfield microscope. Images in Figure 8n,o were captured with an Olympus FV4000 confocal microscope. Images in Figure 9 were captured using a Zeiss 900 confocal microscope.

### Manual Microscopy Navigation

User navigates samples manually using the µCodes grid (Figure 5a). Target relocation was accomplished through sequential navigation along grid coordinates (Figure 5b) or through alternating movements in x and y directions (Figure 5c). To generate the sequential imaging data reported here, after the initial imaging and identification of the samples/targets of interest and the closest µCode to the identified target, the samples were transferred to be imaged under high resolution confocal microscopy or scanning electron microscopy. For either of these, after loading the samples, focus was first directed to the substrate. The nearest µCode was then located and the location was decoded. The neighboring µCodes were followed in ascending or descending order in x and y directions to reach the closest µCode to the target identified during the first imaging session. The filter structures and the cells were then located relative to their closest µCode.

### Navigation Application

The App design module in MATLAB R2023a was utilized to develop the navigation app. The MATLAB application was developed that establish the coordinate relationship between the instrument positioning system and the µCodes reference frame (Figure 5e). The software requires users to locate just two arbitrary µCodes and input their coordinates, from which it calculates transformation parameters and substrate scaling factors (Figure 5d). The application then guides users to the desired targets by calculating their locations (Figure 5f), accommodating non‐uniform substrate deformations that may occur during sample preparation. The mathematical relations behind the coordinate mapping are described in the supporting information. The mathematical relations are available in MATLAB, Phyton, and C++ code format here: github.com/Arefvp/MicroCodes/tree/main/Navigation.

### Electron Microscopy

After the immunofluorescence staining and imaging, the devices were transferred to a filter holder and the cells were incubated with Osmium tetroxide and tannic acid for 60 min each. The sample was then incubated in gradual increases of Ethanol (70, 80, 90, and 100%) for 10 min for each step. The cells were then dried using HMDS for 5 min followed by leaving at room temperature for 30 min. After chemical processing, 10 nm of Au/Pd with a 60/40 ratio was deposited on the cells to reduce charging during imaging. The µCoded‐slides were coated 10 nm of Au/Pd with a 60/40 ratio before imaging. All SEM images were taken using a Hitachi SU8230 FE‐SEM.

### Image Processing

ImageJ and Inkscape were used for image processing. Except for the overlaying images, all other images were only cut and/or rotated to fit the figure. For the overlaying images, one image was set as a reference and the other was scaled, rotated, and cut to make the µCodes align with the corresponding ones in the reference image. For instance, where multiple channels of color exist, all the images of the set were grouped together with the Brightfield/ T‐PMT(transmitted light) image on top. The images were then all transformed as a group. Finally, the overlaying image was aligned with the reference image. For the cell‐level images, the cell borders and features were aligned instead to compensate for the cell shrinkage during chemical processing for EM. The transparency of the top image was set to 50% to make the final composite image.

## Conflict of Interest

Georgia Tech has filed for patent protection covering the navigation aspect of the technology described in this paper. A.V. is the recipient of the Georgia Tech Office of Technology Licensing GT Next award aimed at assisting for commercialization of the described technology.

## Author Contributions

A.V. and J.H. conceptualized and designed the grid system. S.N.H. prepared the cell and tissue samples. A.V., J.H., and S.N.H. performed the experiments. A.V. developed the platform, fabricated the devices, and performed the image processing. A.V. and S.N.H. designed the coded culture slides and dishes, designed the imaging experiments and applications, and wrote the first draft. All authors reviewed, edited, and approved the final manuscript.

## Supporting information



Supporting Information

Supplemental Movie 1

Supplemental Movie 2

## Data Availability

The data that support the findings of this study are available from the corresponding author upon reasonable request.
