# Supplementary material for: µCodes: A Universal Grid Platform for Microscale Mapping, Microscopy Navigation, and Multimodal Imaging
Source: Small. 2025 Aug 21;21(51):e06183. doi: 10.1002/smll.202506183 (PMC12723346; doi:10.1002/smll.202506183)
Supplement: Supplementary file 1 — Supporting Information [file SMLL-21-e06183-s003.pdf]

NANO · MICRO
small

Supporting Information

for *Small*, DOI 10.1002/smll.202506183

μCodes: A Universal Grid Platform for Microscale Mapping, Microscopy Navigation, and Multimodal Imaging

Aref Valipour, Jungmin Ha and Stephen N. Housley**

Supplementary Information for

μCodes: A Universal Grid Platform for Microscale Mapping, Microscopy Navigation, and Multimodal Imaging

Aref Valipour[1, 2, 3, *], Jungmin Ha[1, 4, 5], Stephen N. Housley[1, 5, 6,*]

[1]School of Biological Sciences, Georgia Institute of Technology, Atlanta, Georgia, USA

[2]School of Electrical and Computer Engineering, Georgia Institute of Technology, Atlanta, Georgia, USA

[3]Scheller College of Business, Georgia Institute of Technology, Atlanta, Georgia, USA

[4]George W. Woodruff School of Mechanical Engineering, Georgia Institute of Technology, Atlanta, Georgia, USA

[5]Institute for Bioengineering and Biosciences, Georgia Institute of Technology, Atlanta, Georgia, USA

[6]Winship Cancer Institute, Emory University, Atlanta, GA, USA

A.V. and J.H. contributed equally to this work.

*Correspondence to: Aref@gatech.edu, NickHousley@gatech.edu

Keywords: μCodes, MicroCodes, Multi Modal Imaging, Cell Characterization, Electron Microscopy, Imaging Grids

The mathematical correlation of instrument and the μCodes coordinates where μCode $_1(u_1, v_1)(x_1, y_1)$ and μCode $_2(u_2, v_2)(x_2, y_2)$ are given as two alignment μCodes. $\theta,\ a, and\ b$ are calculated transfer function parameters. (x_t, y_t) is the calculated location for any given target μCode $_t(u_t, v_t)$.

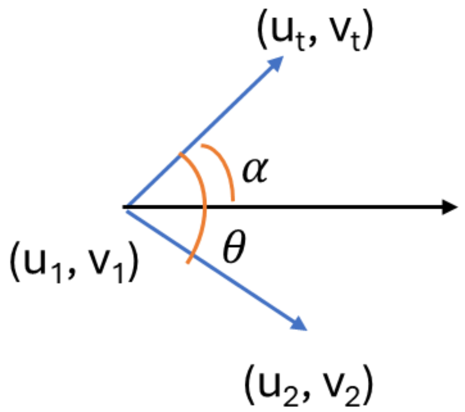

$$\theta = tg^{-1}\left(\frac{v_t - v_1}{u_t - u_1}\right) - tg^{-1}\left(\frac{v_2 - v_1}{u_2 - u_1}\right)$$

$$\alpha = \theta - tg^{-1}\left(\frac{y_2 - y_1}{x_2 - x_1}\right)$$

$$R_{t,xy} = \sqrt{(u_t - u_1)^2 + (v_t - v_1)^2}\ \frac{\sqrt{(x_2 - x_1)^2 + (y_2 - y_1)^2}}{\sqrt{(u_2 - u_1)^2 + (v_2 - v_1)^2}}$$

$$x_t = R_{t,xy}\ .Cos(\alpha)$$

$$y_t = R_{t,xy}\ .Sin(\alpha)$$

Supplementary Table 1: Comparison of μCodes design and footprint

μCode Variant	*# of Bits*	*Numeric Range*	*Minimum Feature Size*	*Footprint*			
			(μm)	x (μm)	y (μm)	Max(x,y)	Area (μm^2)
7-Bit Binary	7	(0, 127)	1	8	4	8	32
	7	(0, 127)	2	16	8	16	128
	7	(0, 127)	5	40	20	40	800
	7	(0, 127)	10	80	40	80	3200
	7	(0, 127)	20	160	80	160	12800
10-Bit Binary	10	(0, 1023)	1	11	4	11	44
	10	(0, 1023)	2	22	8	22	176
	10	(0, 1023)	5	55	20	55	1100
	10	(0, 1023)	10	110	40	110	4400
	10	(0, 1023)	20	220	80	220	17600
Numerical	3	(0, 999)	1	35	7	35	245
	3	(0, 999)	2	70	14	70	980
	3	(0, 999)	5	175	35	175	6125
	3	(0, 999)	10	350	70	350	24500
	3	(0, 999)	20	700	140	700	98000
Ternary	5	(0,728)	1	42	10	42	420
	5	(0,728)	2	84	20	84	1680
	5	(0,728)	5	210	50	210	10500
	5	(0,728)	10	420	100	420	42000
	5	(0,728)	20	840	200	840	168000
PLANET	10	(0, 1023)	1	44	10	44	440
	10	(0, 1023)	2	88	20	88	1760
	10	(0, 1023)	5	220	50	220	11000
	10	(0, 1023)	10	440	100	440	44000
	10	(0, 1023)	20	880	200	880	176000

Supplementary Table 2: Retention of targets (HEY-A8 cells) when using µCoded filters for following the cells through confocal microscopy and SEM. Each cell was observed for changes that happened through the imaging pipeline. Cells were labeled "Stayed" if they were in the exact same location in both the confocal and SEM image, "Moved" if they moved within the well, and "Not Found" if the cell was not detectable in the SEM image. This data suggests that while some cells were lost in the chemical preparation process for SEM, the majority of cells survived the process and stayed in the same location. This experiment did not account for the cell shrinkages.

	STAYED	MOVED	NOT FOUND
**OBSERVATION 1**	25	5	0
**OBSERVATION 2**	28	5	5
**OBSERVATION 3**	5	1	5
**TOTAL**	58	11	10
**% OF ALL**	%73.4	%13.9	%12.7

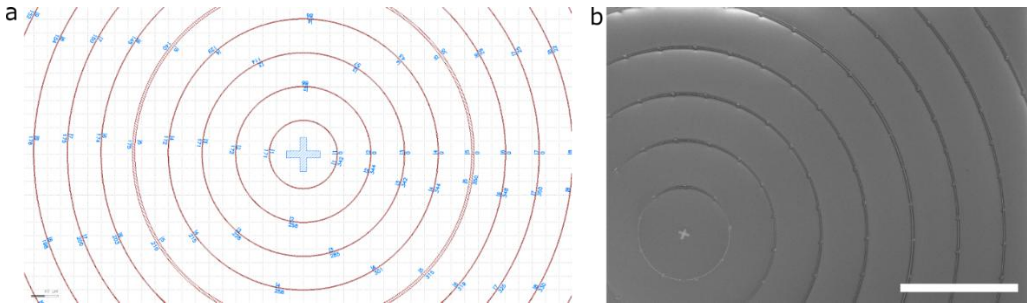

Supplementary Figure 1: a An schematic of μCodes utilizing polar coordinates and Numerical digit. **b** A SEM image of fabricated polar μCodes. The scale bar is 500 microns.

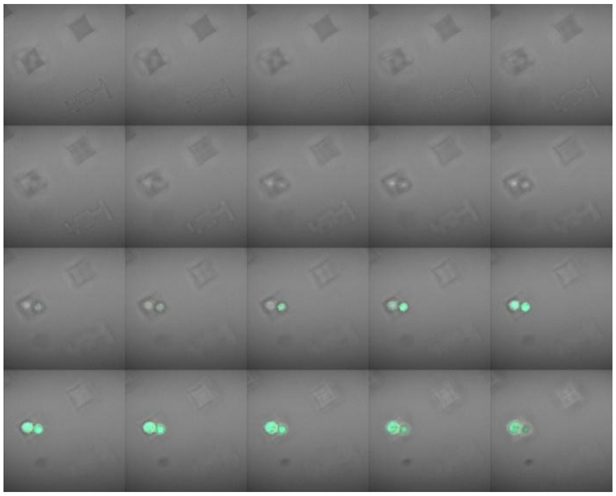

Supplementary Figure 2: Z-stack image of μCodes alongside the target cells. The μCodes are designed to be on a different height than the cells to prevent light interference.

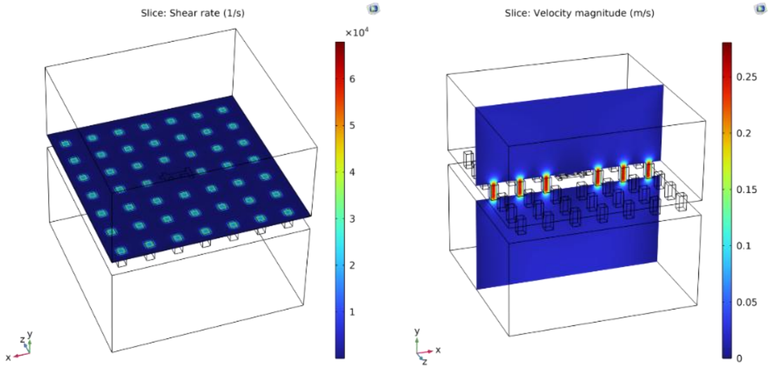

Supplementary Figure 3: COMSOL simulations demonstrate the velocity magnitude, and the shear fields of the filter structures embedded with the μCodes. The results suggest that the μCodes are not interfering with the flow profile and hence the operations of the filters.

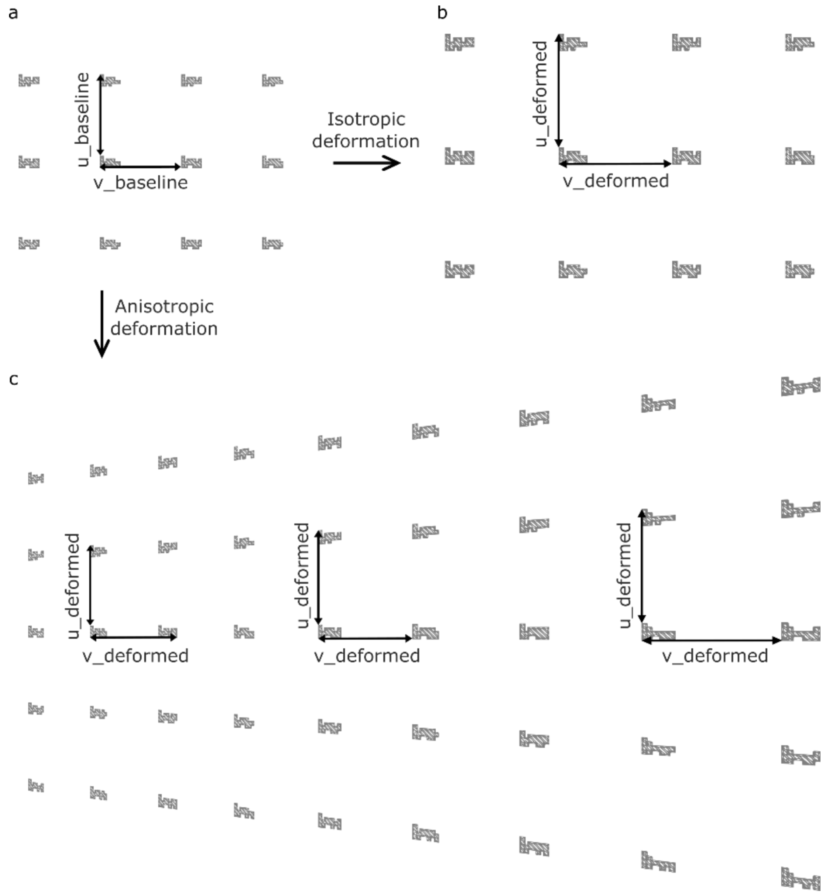

Supplementary Figure 4: Theoretical logic of μCodes compensation for distortion. In devices embedded with μCodes made with material that deform under changing conditions, e.g. expand or shrink due to change in temperature or water absorption, the μCodes will deform at the same rate as the material. In both isotropic and anisotropic deformation, μCodes can be relied on as a reference for deformation. **a** illustrates a base line structure before deformation, **b** the same structure under isotropic deformation, and **c** the structure deformed under anisotropic conditions.

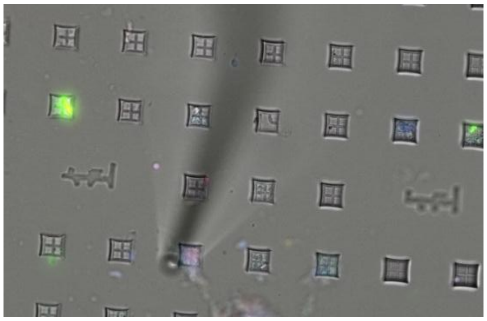

Supplementary Figure 5: An example of utilizing µCodes for target mapping in micromanipulation and picking cells of interest for downstream analysis. Visible as a dark silhouette against the background, a microneedle can be seen performing the cell extraction procedure. The tip of the microneedle is seen on the filter surface.
